# Chemical Uniformity in Ferroelectric K*_x_*Na_1−_ *_x_*NbO_3_ Thin Films

**DOI:** 10.1002/gch2.201800114

**Published:** 2019-08-01

**Authors:** Henrik H. Sønsteby, Ola Nilsen, Helmer Fjellvåg

**Affiliations:** ^1^ Department of Chemistry University of Oslo 0315 Oslo Norway

**Keywords:** atomic layer deposition, functional perovskites, lead‐free ferroelectrics, thin films

## Abstract

Potassium sodium niobate (KNN) has long been considered a viable candidate for replacing lead‐based materials in piezo‐ and ferroelectric devices. The introduction of KNN on an industrial scale is highly awaited; however, processing challenges still remain to be solved. The main obstacle is lack of reproducible growth of uniform boules or thin films at temperatures that facilitate monolithic device integration. Herein, atomic layer deposition (ALD) of KNN thin films, exhibiting high chemical uniformity over large areas, is reported. The cation composition can be controlled at a 1% level, enabling fine‐tuning of the film stoichiometry across the morphotropic phase boundaries of the KNbO_3_–NaNbO_3_ solid solution. The films are obtained as highly oriented on Pt (111)||Si (100)‐substrates after annealing at temperatures as low as 550 °C. They exhibit converse piezoelectric effects with magnitudes in accordance with literature. It is believed that the successful development of the described ALD process represents a major step toward achieving lead‐free piezo‐ and ferroelectrics on an industrial scale.

## 1 Introduction

Piezo‐ and ferroelectrics are an integral part of modern technological society, with major applications in sensor and actuator technology, and emerging applications in nonvolatile memory, bandpass filters and imaging technology.^1^, ^2^ The world market is estimated to have passed $20 billion in 2018, and is steadily increasing. Current materials of choice are lead based perovskites, commonly PbZr_1−_ *_x_*Ti*_x_*O_3_ (PZT) and PbMg_1/3_Nb_2/3_O_3_‐PbTiO_3_ (PMN‐PT). The popularity of these materials stems from facile preparation, low cost, highly applicable physical properties, and high tunability.^3^, ^4^ Their properties can be selectively varied by altering cation stoichiometry and/or doping, thereby fine‐tuning to specific demands and applications.^3^, ^5^, ^6^, ^7^, ^8^ They are easily poled, they have high Curie temperatures (*T*_c_) and withstand harsh environments. Their only flaw, which is a major one, is their large content of some 60 wt% of lead (Pb).

The acute toxicity of lead has been known for centuries, however, recent studies have shown that bodily accumulation is dangerous even at very low concentrations. The accumulation is most pronounced for children and carrying women. For Americans, elevated lead levels is estimated to be an attributable mortality factor for more than 400 000 deaths annually.^9^ Such concerns are highly relevant for the working conditions and toxic local environment at lead smelters in producing countries.^10^

Since several years, there has been a strong push for removing lead in consumer products. The directive 2002/95/EC (RoHS1) introduced a total ban on lead based materials in electronic consumer products within the European Union (EU). There are, however, restriction exemptions for the use of lead when no other viable option exists. Since many electronic products require a piezo‐ or ferroelectric component, there is still a generous amount of lead in widely used consumer products.

The toxicity of lead and the strict legislation has triggered a surge for finding alternative materials with properties that match lead based piezo‐ and ferroelectrics.^11^ These should fulfil several requirements: high applicable Curie temperature (*T*_c_ > 300 °C), high piezoelectric coefficient (*d*_33_); and existence of a morphotropic phase boundary (MPB) around which key functional properties are enhanced. Very few lead free materials fulfill these criteria. The most studied are the bismuth sodium titanate‐ (Bi_1/2_Na_1/2_TiO_3_, BNT) and potassium sodium niobate‐ (K*_x_*Na_1−_ *_x_*NbO_3_, KNN) type systems. BNT is a relaxor type ferroelectric, so the description of a MPB is somewhat controversial, hampering its usability.^12^ KNN suffers from a slightly low piezoelectric response (*d*_33_ ≈ 120 pC N^−1^), but has an applicable *T*_c_ (≈420 °C) and a sharp MPB at approximately K_0.5_Na_0.5_NbO_3_ at the phase transition between antiferroelectric and ferroelectric orthorhombic structures. Note that this MPB is the one commonly referred to and the one of current focus, however, two more MPBs exist in the KNbO_3_‐NaNbNO_3_ solid solution system.

The possibility to enhance the piezoresponse of KNN by selective substitution is an ongoing worldwide effort. So far, one has succeeded to more than triple *d*_33_, reaching applicable values (*d*_33_ > 400 pC N^−1^) for lithium‐, antimony‐, barium‐, zirconium‐, and titianium‐containing variants.^11^

KNN is considered as a viable replacement of PZT at lab scale. However, it is not yet embraced by industry. This is mainly due to difficulties in the synthesis of homogeneous crystals and/or films. Typical crystal growth produces boules of limited size (<5 cm) and with large stress induced zone boundaries throughout the boule. More problematic, the cation composition is hard to control throughout the growth process, leading to gradients in the K/Na‐composition and thereby nonuniform piezoresponse. The current approach is to produce large boules, and then cut out a crystal with the correct stoichiometry, which is tedious, ineffective and expensive.

Deposition of thin films of KNN has been as achieved by pulsed layer deposition (PLD), metal–organic chemical vapor deposition (MOCVD), and sol–gel coating.^13^, ^14^, ^15^, ^16^ PLD typically requires elevated growth temperatures (>600 °C) with challenges in control of cation composition. Uniform, single‐deposition PLD growth over larger areas (>10 cm) remains yet to be overcome. MOCVD growth suffers from high growth and/or annealing temperatures (>800 °C), which make monolithic integration on functional devices difficult. Controlling the K:Na‐uniformity is likewise challenging. Sol–gel methods may provide relatively uniform films over larger areas. Unfortunately, these tend to suffer from precursor pre‐reactions, from crack formation due to shrinkage during solvent evaporation as well as carbon contamination often being detrimental to functionality.

Atomic layer deposition (ALD) is a layer‐by‐layer self‐limiting thin film growth technique that offers high chemical uniformity and thickness conformality over large areas.^17^ It has traditionally been used to deposit high‐κ binary oxides and nitrides for microelectronics applications, and is implemented in industry for ultra‐small node transistors in processing units. The available chemistry for ALD has increased strongly over the last years, and the technique can now be used for depositing high‐quality thin films of complex oxides.^18^, ^19^ The technique is often regarded slow, but uniform coverage in large reaction chambers (2D and 3D) allow for deposition on many devices at the same time, making the total throughput viable on an industrial scale.

ALD was recently used to deposit LiNbO_3_ with decent functional properties.^20^ With the advent of useful sodium‐ and potassium containing ALD precursors, it is now possible also to obtain *K_x_*Na_1−_ *_x_*NbO_3_ films using similar procedures.^21^, ^22^

With this study, we have applied ALD to grow thin films of KNN with very high chemical conformality at temperatures as low as 250 °C. The films are stoichiometrically uniform as grown in a 30 cm chamber, proving the ability of successful deposition over large areas, i.e., for many devices. The cation stoichiometry is tuned at a 1% level, giving sufficient control to obtain compositions close to the MPB. The current growth rate of 100 nm h^−1^ is likely to benefit further from future optimization. The process is highly reproducible, and easily implemented in batch processing at industry scale. These findings hopefully represent a giant leap toward abolishing the lead dominance of piezo‐ and ferroelectrics.

## 2 Results and Discussion

### 2.1 Conformal Growth

The deposition of complex oxides by ALD is normally carried out by combining binary oxide processes to form a supercycle process. In the case of alkali metal containing films, this is not as straightforward, since the alkali metal binary oxides are not stable. In other words, the stoichiometric ratios between Na/K and Nb must be tuned simultaneously. This is carried out by growing films with gradually increasing alkali metal content, while monitoring the deposited Na/K to Nb cation ratio. In this study, we focused on the stoichiometric 1:1 ratio between alkali metal and niobium in correspondence with the wanted perovskite type phase. As the structure and chemistry of the two precursor systems (*tert*‐butoxide vs ethoxide) is very different, the 1:1 ratio is not achieved at a 1:1 pulsed ratio between the precursors, but at a 2:7 ratio between *A*O*^t^*Bu (*A* = Na, K) and Nb(OEt)_5_. The applied process is described by

(1) $$n\times \left(\begin{array}{l} 3\times \left[\mathrm{Nb}{\left(\mathrm{OEt}\right)}_{5}+H_{2}O\right]+AO^{t}\mathrm{Bu}+H_{2}O+\\ 4\times \left[\mathrm{Nb}{\left(\mathrm{OEt}\right)}_{5}+H_{2}O\right]+AO^{t}\mathrm{Bu}+H_{2}O \end{array}\right)$$

Here, *n* describes the full supercycle, and the film thickness is determined by *n* alone. We then studied the growth parameters as a function of the ratio between Na and K in *A*O*^t^*Bu (A = Na, K). The latter two precursor systems are very similar, and we expect a close to linear relationship in the deposited concentration of mixtures of the two alkali metal ions. This was investigated by depositing films of K*_x_*Na_1−_ *_x_*NbO_3_ (0 < *x* < 1), with focus on the range around the mentioned MPB (i.e., close to 50%).

**Figure 1:**
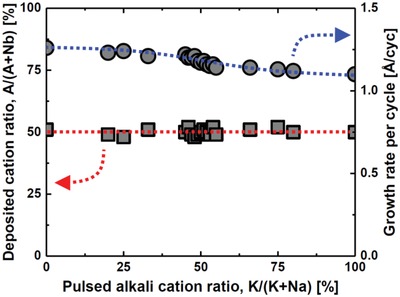
Left *y*‐axis: Deposited cation ratio (A/(A+Nb), A = Na+K) as a function of the pulsed alkali cation ratio (K/K+Na). The red dotted line marks the stoichiometric ANbO_3_ composition. Error bars are within data points. Right *y*‐axis: Growth rate per cation cycle as a function of pulsed alkali cation ratio (K/K+Na). Error bars are within data points. The blue dotted line is a guide‐of‐eye line to emphasize variation.

**Figure 2:**
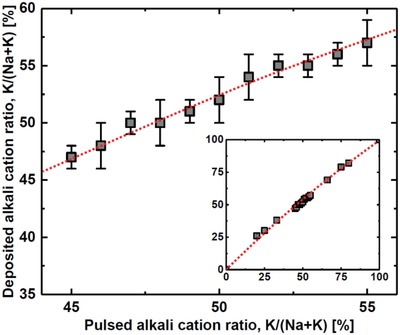
Deposited alkali cation ratio (K/(Na+K)) as a function of the pulsed alkali cation ratio (K(Na+K)) in a composition range close to the morphotropic phase boundary. Inset: Deposited alkali cation ratio (K/(Na+K)) as a function of the pulsed alkali cation ratio (K(Na+K)) for the full composition range (KNbO_3_ → NaNbO_3_). Error bars are within data points. The red dotted lines are guide‐to‐the‐eye to emphasize the variation.

Two essential parameters require attention; the *A*:Nb ratio as the Na:K pulsed ratio in *A* is varied; and the deposited Na:K ratio during the same variation (**Figures** **1** and **2**). From X‐ray fluorescence (XRF) analysis it was evident that the *A*:Nb‐ratio is close to constant, showing that the two *tert*‐butoxide precursors affect the growth of niobium oxide equally. This also means that the *A*:Nb pulsed ratio does not need to be changed when varying the pulsed Na:K ratio, thereby providing straightforward tunability of the Na:K ratio in the achieved films. The growth per cycle is slightly lower for high K‐concentration, without this affecting the overall composition.

Considering the pulsed versus deposited concentration of Na and K in *A*, we observe the expected (close to) linear trend over the whole composition range, see insert to Figure 2. The slight convex curve originates from a small difference in growth per cycle between the (imagined) binary sodium and potassium oxide systems. A detailed study was done for pulsed Na:K compositions close to 50:50, Figure 2. The linear trend represents an important result as it shows that the composition can be tuned at the percent level and thereby to control the Na:K ratio very close to the MPB. We describe below detailed results for the 50:50 deposited samples as achieved by using a 48:52 pulsed K:Na‐ratio.

**Figure 3:**
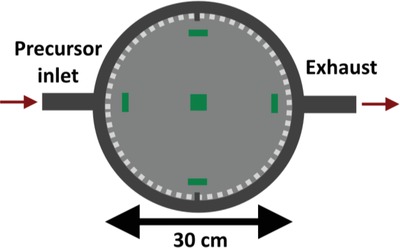
Cartoon of the *Ø* = 30 cm reaction chamber utilized in this study. Substrates are placed according to the green marks.

A limiting factor for most thin film deposition technique is lack of the ability to maintain cation uniformity over a large deposited area. Such abilities are obviously very important when considering potential industrial upscaling. The larger the effective uniform area provided by the process; the more devices can be coated at any given time. We have quantified the compositional homogeneity by utilizing the whole ALD reaction chamber (*Ø* = 30 cm), followed by mapping of the Na:K‐ratio for substrates situated at five different locations in the chamber (**Figure** **3**).

The cation stoichiometry of the films was determined by XRF and X‐ray photoelectron spectroscopy (XPS). XPS had a small probe size of 3 mm, allowing for a quite detailed geometrical mapping. XPS was currently used to quantify the relative ratio between Na and K, whereas XRF was used to determine the stoichiometry on an absolute scale.

**Figure 4:**
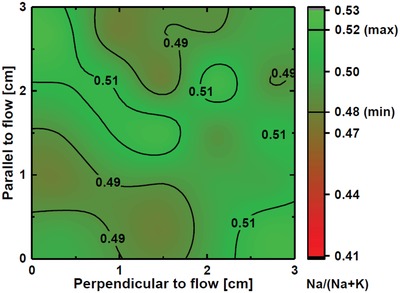
Map of the Na/(Na+K) composition ratio on the central substrate for a 52:48 Na:K pulsed cation ratio, as measured by X‐ray photoelectron spectroscopy. Color map is chosen to emphasize applicable functional properties (*x* < 40 is unacceptable). Maximum variation in the sample is 0.48 < *x* < 0.52.

**Figure** **4** shows a map of the Na:K cation ratio distribution over a 3 × 3 cm^2^ substrate placed in the middle of the chamber. The ratio is measured at 25 different locations, spaced minimum 6 mm apart. The stoichiometry measured by XPS varies between K_0.48_Na_0.52_NbO_3_ and K_0.52_Na_0.48_NbO, with all points within 2σ of K_0.50_Na_0.50_NbO_3_ $\left(\sigma =\sqrt{\frac{1}{N}\sum_{i=1}^{N}x_{i}-\mu }=0.014\right)$. As there is no evident directional dependence, we assume that the variation represents uncertainty in the measurements, rather than being a physical manifestation. In any case, the variation is so small that the functional properties over the observed compositional interval will be close to equal, i.e., we expect Δ*d*_33_ < 1%.^23^

**Figure 5:**
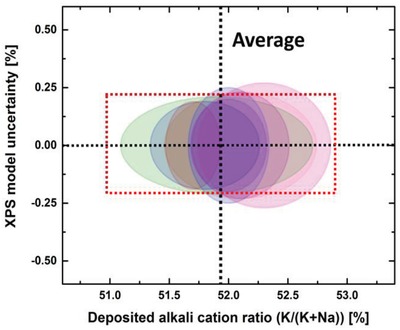
Eight consecutive depositions using exactly the same parameters (with Nb_2_O_5_ passivation in between every deposition). Ellipses show the variation in deposited alkali ratio (K/(K+Na), maximum across all directions in the chamber, *x*‐axis), and the uncertainty in the XPS‐model, *y*‐direction. The dimensions of the red box illustrate the expected variation in deposited stoichiometry by adding one additional pulse of one of the alkaline elements, *x*‐direction, and the instrumental uncertainty of the XPS system, *y*‐direction.

The total variation across the whole reaction chamber is shown in **Figure** **5** based on eight depositions. The ellipses are constructed by taking the maximal cation variation (*x*‐direction) and the uncertainty in the XPS quantification (*y*‐direction). The eight depositions were done with the exact same parameters (pulsed K:Na = 50:50) in order also to investigate reproducibility. The maximum variation across all directions in the 30 cm chamber is ≈2 cat% when comparing eight depositions. This is well within acceptable values in terms of functionality. The obtained average value is 51.99 ± 0.55 cat% Na. We estimated the expected variation in cation stoichiometry if replacing one Na‐pulse with a K‐pulse or vice versa (red dotted box in Figure 5). This shows that the stoichiometry of the deposited films is highly reproducible, and that the cation concentration can be fine‐tuned on the single pulse level.

Note that a passivation deposition step of 22 nm Nb_2_O_5_ (500 cycles of Nb(OEt)_5_ + H_2_O) was added in between every KNN‐iteration, to avoid memory effects from the preceding deposition.^22^ This slightly slows down any batch processing, however, still two depositions of micrometer thickness could be facilitated per day, per reactor. We expect that significant process effectivization can be achieved by optimizing precursor temperature and pulse‐ and purge times.

### 2.2 Structure and Functionality

The structure and functionality were studied of uniform films, K_0.52_Na_0.48_NbO_3_ deposited on Pt(111)||Si(100). The Pt(111) surface facilitates epitaxial growth of KNN. Best lattice match is achieved for the slightly shorter *a* and *b* lattice parameters, hence we expect tetragonal (or orthorhombic but very close to tetragonal) KNN with the longer *c*‐axis predominantly oriented perpendicular to the substrate plane (KNN(001)|[100]||Pt(111)|Pt($\bar{1}$10||Si(100)|Si(001).

The obtained films are X‐ray amorphous as deposited at 250 °C, which is typical for low‐temperature chemically deposited thin films. Crystallization was achieved by postannealing at 550 °C for 30 min. The required annealing temperature is low compared to treatments required for other techniques, and will facilitate monolithic integration for most applications.

**Figure 6:**
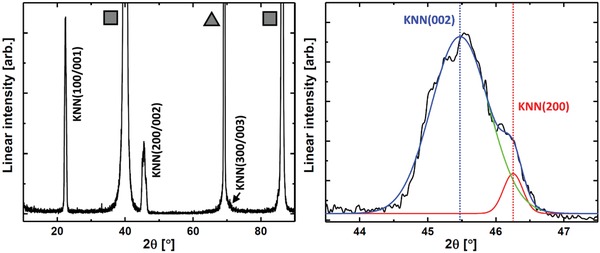
Left: X‐ray diffractogram of 120 nm K_0.52_Na_0.48_NbO_3_ on Pt(111)@Si(100). Pt‐reflections are marked by grey squares, the Si(400)‐reflection is marked by a gray triangle. Right: Fitted (002/200) reflection of K_0.52_Na_0.48_NbO_3_. Red line corresponds to KNN(200), green line corresponds to KNN(002). Blue line is the total fit.

X‐ray diffraction (XRD) shows sharp reflections from oriented KNN, as well as from Pt(111) and Si(100) (**Figure** **6**a). No other reflections are observed, which indicates that the film is fully oriented out‐of‐plane, and that no secondary crystalline phases are present. However, taking a closer look at (002) it is evident that it is at least split in two. A two Gaussian model gives the best fit for the reflection, however we cannot rule out that the films are orthorhombic and that a third reflection is present. These two reflections correspond to KNN(002) and KNN(200), with a difference in unit cell parameters of ≈0.05 Å, in accordance with expectations. A corresponding split is not observed for the KNN(100/001)‐peak due to resolution limitations. The weighted (integrated) area after peak deconvolution of KNN(002) and KNN(200) indicates a 1:15 ratio, meaning that ≈7% of the KNN crystallites are (100)‐oriented. This is not detrimental to function, however, the situation may nonetheless possibly be avoided by post‐annealing in an electric field.^24^

**Figure 7:**
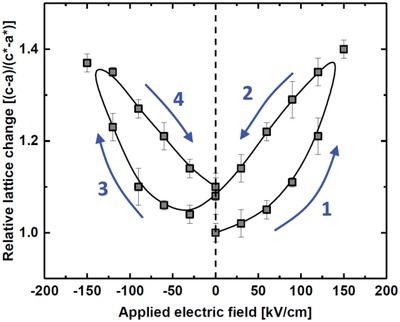
Degree of tetragonal distortion in 120 nm K_0.52_Na_0.48_NbO_3_ on Pt(111)@Si(100) as a function of applied electric field, as measured by in situ synchrotron XRD. The sample was unpoled at the beginning of the experiment. Arrows mark the direction in which the field was varied. The black line is a fitted *b*‐spline function of the measured data.

We probed the piezo‐/ferroelectric functionality of the KNN films by using in situ XRD (at SNBL@ESRF, Grenoble, France) upon perturbating the material by means of an electric field. The setup is described in a previous publication.^25^ This allows careful identification of the *c‐* and *a‐*axis of the cell under electric load. The bottom Pt‐layer is contacted in a corner of the specimen that was masked during deposition and hence works as the bottom electrode. Gold contacts are deposited on the surface of the KNN film, working as top contacts. **Figure** **7** shows the relative change in unit cell parameters in terms of degree of tetragonal distortion as a function of applied electric field. We observe a major, ≈40%, change in the degree of tetragonal distortion, as well as hysteresis on increasing and decreasing fields (coercive field, *H*_c_ ≈ 50 kV cm^−1^). The latter crude estimate is higher than bulk literature values, however, this is not uncommon for thin film ferroelectrics due to domain wall pinning and strain‐related effects.^26^, ^27^, ^28^

The change in tetragonal distortion upon perturbation by an electric field can be transformed into an estimate of the converse piezoelectric coefficient, by taking only the strain induced component along the *c*‐axis into account. When the field is applied perpendicular to the surface and strain is measured along the KNN *c*‐axis, the generalized equation for the induced strain

(2) $$\left[\begin{array}{l} \varepsilon_{1}\\ \varepsilon_{2}\\ \varepsilon_{3}\\ \varepsilon_{4}\\ \varepsilon_{5}\\ \varepsilon_{6} \end{array}\right]=\left[\begin{array}{lll} d_{11} & d_{21} & d_{31}\\ d_{12} & d_{22} & d_{32}\\ d_{13} & d_{23} & d_{33}\\ d_{14} & d_{24} & d_{34}\\ d_{15} & d_{25} & d_{35}\\ d_{16} & d_{26} & d_{36} \end{array}\right]\left[\begin{array}{l} E_{1}\\ E_{2}\\ E_{3} \end{array}\right]\overset{\mathrm{Tetragonal}\;\mathrm{KNN}}{\to }\left[\begin{array}{lll} 0 & 0 & d_{31}\\ 0 & 0 & d_{31}\\ 0 & 0 & d_{31}\\ 0 & d_{15} & 0\\ d_{15} & 0 & 0\\ 0 & 0 & 0 \end{array}\right]\left[\begin{array}{l} E_{1}\\ E_{2}\\ E_{3} \end{array}\right]$$

with *ε_j_* being strain, *d_ij_* the converse piezoelectric coefficient, and *E_i_* the applied electric field, reduces to a simple relationship:

(3) $$\varepsilon_{3}=d_{33}E_{3}$$

Using the absolute values of the elongation in the *c*‐axis and the potential across one unit cell (averaged to 1.58 millistrain V^−1^ for a series of measurements), the converse piezoelectric coefficient is estimated to *d*_33_ ≈ 105 pm V^−1^ (or pC N^−1^). It should be noted that this is a crude estimate, as variations in the cell‐parameters of magnitude in the 0.001 Å range is close to the resolution limit of the diffraction measurements. The value is nonetheless close to previously reported values for KNN thin films, and we believe this serves as proof‐of‐concept that atomic layer deposited thin films of KNN preserve very attractive functionality.

## 3 Conclusion

Thin films of K*_x_*Na_1−_ *_x_*NbO_3_ with very high structural and chemical uniformity is deposited by atomic layer deposition. In literature, the challenge to receive a uniform K/Na‐ratio throughout single crystals or crystalline films has been considered difficult to overcome. We have currently proved successful deposition of KNN with controlled composition and excellent chemical uniformity on the percent level for a reaction chamber with diameter *Ø* = 30 cm. The uniformity has been reproduced with high accuracy, with only minor variations in chemical composition and thickness. The adopted deposition temperatures are much lower than for competing thin film techniques and the required post‐annealing crystallization was achieved at 550°C, i.e., at temperatures sufficiently low for facilitating monolithic integration into devices. The favorable structural and functional properties of KNN were confirmed through diffraction analysis, both ex situ and in situ under perturbation by an electric field. Based on these experiments we have estimated a converse piezoelectric coefficient *d*_33_ ≈ 105 pm V^−1^, which is in agreement with previously reported values.

In our opinion the results clearly show that the cation uniformity issue can be overcome by using ALD. This opens for further work on chemical substitutions into the KNN matrix to obtain films with enhanced functional properties that may match those of lead‐based piezoelectrics. We consider this as a large step on the way to abolish the lead dominance in piezo‐ and ferroelectrics.

## 4 Experimental Section

Thin films depositions were carried out in a TFS 500 reactor (Beneq Oy). All films were deposited at a reactor temperature of 250 °C, unless otherwise stated. Purging gas was supplied from a Nitrox UHPN 3001 generator producing 99.9995% N_2_, and maintained at a 300 cm^3^ min^−1^ primary flow rate. Reactor operating pressure was maintained at 1.0 mbar throughout the depositions.

All depositions were carried out using potassium *tert*‐butoxide (KO*^t^*Bu, Aldrich, 97%), sodium *tert*‐butoxide (NaO*^t^*Bu, Aldrich, 97%), and niobium ethoxide (Nb(OEt)_5_, Sigma Aldrich, 99%) as metal precursors. The *tert*‐butoxides were supplied from HS 300 metal bubbler sources heated to 150 and 140 °C for KO*^t^*Bu and NaO*^t^*Bu, respectively. Nb(OEt)_5_ was supplied from an open glass boat in a HS 500 source maintained at 68 °C. Deionized H_2_O was used as the oxygen source, supplied from a glass bubbler maintained at room temperature. Pulse times were set to 3, 3, 2, and 0.25 s for KO*^t^*Bu, NaO*^t^*Bu_,_ Nb(OEt)_5_, and H_2_O, respectively. All purge times were set to 2 s. These deposition parameters were chosen to obtain self‐limiting growth as previously reported.^22^

The films were deposited on 1 × 1 and 3 × 3 cm^2^ Si (100) substrates for characterization of thickness, conformality, and composition. Selected films were deposited on Pt(111)||Si(100) (SINTEF, Norway) for characterization of electrical properties.

The thin film thickness was measured using a J. A. Woollam alpha‐SE spectroscopic ellipsometer in the range of 390–900 nm. A Cauchy‐function was successfully used to model the collected data.

Rapid thermal annealing was carried out in an OTF‐1200X (MTI Corp.) furnace heated by halogen lamps. A ramp rate of 10 °C s^−1^ was employed to reach the set temperature.

X‐ray diffraction measurements were performed on a Bruker AXS D8 Discover diffractometer equipped with a LynxEye strip detector and a Ge (111) focusing monochromator, providing Cu Kα_1_ radiation (λ = 1.5406 Å).

In situ determination of unit cell variations under perturbation by an electric field was carried out with synchrotron X‐ray diffraction at the Swiss‐Norwegian Beamlines at the European Synchrotron Radiation Facility (BM01 ‐ SNBL@ESRF) in Grenoble, France. BM01 is a bending magnet end station. A monochromatic beam with wavelength 0.6745 Å was employed. The setup is described previously in literature.^25^

Average chemical composition was analyzed using a Panalytical Axios Max Minerals XRF system equipped with a 4 kW Rh‐tube. The system is running with Omnian and Stratos options for standardless measurements.

Complimentary measurements of composition and spatial cation distribution were carried out with XPS, using a Thermo Scientific Theta Probe Angle‐Resolved XPS system. The instrument is equipped with a standard Al Kα source (*hν* = 1486.6 eV). Pass energy values of 200 and 60 eV were used for survey spectra and detailed scans, respectively. The data was corrected for shifts using the peak from adventitious carbon (binding energy = 284.8 eV) as reference.

## Conflict of Interest

The authors declare no conflict of interest.

## References

[1] N. Setter , D. Damjanovic , L. Eng , G. Fox , S. Gevorgian , S. Hong , A. Kingon , H. Kohlstedt , N. Y. Park , G. B. Stephenson , I. Stolitchnov , A. K. Taganstev , D. V. Taylor , T. Yamada , S. Streiffer , J. Appl. Phys. 2006, 100, 051606.

[2] K. Asif , A. Zafar , K. Heung Soo , O. Il‐Kwon , Smart Mater. Struct. 2016, 25, 053002.

[3] N. Izyumskaya , Y. I. Alivov , S. J. Cho , H. Morkoç , H. Lee , Y. S. Kang , Crit. Rev. Solid State Mater. Sci. 2007, 32, 111.

[4] Z.‐W. Yin , H.‐S. Luo , P.‐C. Wang , G.‐S. Xu , Ferroelectrics 1999, 229, 207.

[5] N. A. Pertsev , V. G. Kukhar , H. Kohlstedt , R. Waser , Phys. Rev. B 2003, 67, 054107.

[6] Q. Zhang , R. W. Whatmore , J. Appl. Phys. 2003, 94, 5228.

[7] V. Kayasu , M. Ozenbas , J. Eur. Ceram. Soc. 2009, 29, 1157.

[8] P. K. Panda , B. Sahoo , Ferroelectrics 2015, 474, 128.

[9] B. P. Lanphear , S. Rauch , P. Auinger , R. W. Allen , R. W. Hornung , Lancet Public Health 2018, 3, e177. 29544878 10.1016/S2468-2667(18)30025-2

[10] J. Watts , Lancet 2009, 374, 868. 19757511 10.1016/s0140-6736(09)61612-3

[11] C.‐H. Hong , H.‐P. Kim , B.‐Y. Choi , H.‐S. Han , J. S. Son , C. W. Ahn , W. Jo , J. Materiomics 2016, 2, 1.

[12] E. Sapper , N. Novak , W. Jo , T. Granzow , J. Rödel , J. Appl. Phys. 2014, 115, 194104.

[13] H. Scott , M. Paul , IOP Conf. Ser.: Mater. Sci. Eng. 2010, 8, 012004.

[14] W. Zhou , F. Liu , C. He , J. Chen , presented at 2012 Symp. Photonics and Optoelectronics, Shanghai, China, May 2012.

[15] G. H. Khorrami , A. Kompany , A. Khorsand Zak , Mater. Lett. 2013, 110, 172.

[16] R. S. Deol , M. Mehra , B. Mitra , M. Singh , MRS Adv. 2018, 3, 269.

[17] S. M. George , Chem. Rev. 2010, 110, 111. 19947596 10.1021/cr900056b

[18] V. Miikkulainen , M. Leskelä , M. Ritala , R. L. Puurunen , J. Appl. Phys. 2013, 113, 021301.

[19] H. H. Sønsteby , H. Fjellvåg , O. Nilsen , Adv. Mater. Interfaces 2017, 4, 1600903.

[20] E. Østreng , H. H. Sønsteby , T. Sajavaara , O. Nilsen , H. Fjellvåg , J. Mater. Chem. C 2013, 1, 4283.

[21] E. Østreng , H. H. Sønsteby , S. Øien , O. Nilsen , H. Fjellvåg , Dalton Trans. 2014, 43, 16666. 25265332 10.1039/c4dt01930j

[22] H. H. Sønsteby , O. Nilsen , H. Fjellvåg , J. Vac. Sci. Technol., A 2016, 34, 041508.

[23] B.‐P. Zhang , J.‐F. Li , K. Wang , H. Zhang , J. Am. Ceram. Soc. 2006, 89, 1605.

[24] D. Alikin , A. Turygin , A. Kholkin , V. Shur , Materials 2017, 10, 47. 10.3390/ma10010047 PMC5344613 28772408

[25] H. H. Sønsteby , J. Wind , M. Jensen , T. A. Storaas , D. Chernyshov , H. Fjellvåg , Ferroelectrics 2018, 537, 20.

[26] N. Bassiri‐Gharb , I. Fujii , E. Hong , S. Trolier‐McKinstry , D. V. Taylor , D. Damjanovic , J. Electroceram. 2007, 19, 49.

[27] M. D. Biegalski , D. H. Kim , S. Choudhury , L. Q. Chen , H. M. Christen , K. Dörr , Appl. Phys. Lett. 2011, 98, 142902.

[28] K. J. Choi , M. Biegalski , Y. L. Li , A. Sharan , J. Schubert , R. Uecker , P. Reiche , Y. B. Chen , X. Q. Pan , V. Gopalan , L.‐Q. Chen , D. G. Schlom , C. B. Eom , Science 2004, 306, 1005. 15528439 10.1126/science.1103218

